# Frequency-dependent alterations in functional connectivity in patients with Alzheimer’s Disease spectrum disorders

**DOI:** 10.3389/fnagi.2024.1375836

**Published:** 2024-03-28

**Authors:** Hanjun Hu, Luoyu Wang, Sammad Abdul, Xue Tang, Qi Feng, Yuzhu Mu, Xiuhong Ge, Zhengluan Liao, Zhongxiang Ding

**Affiliations:** ^1^The Fourth Clinical College, Zhejiang Chinese Medical University, Hangzhou, China; ^2^Department of Radiology, Hangzhou First People's Hospital, Hangzhou, China; ^3^School of Biomedical Engineering, Shanghai Tech University, Shanghai, China; ^4^International Education College, Zhejiang Chinese Medical University, Hangzhou, China; ^5^School of Medical Imaging, Hangzhou Medical College, Hangzhou, China; ^6^Department of Psychiatry, Zhejiang Provincial People’s Hospital/People’s Hospital of Hangzhou Medical College, Hangzhou, China

**Keywords:** Alzheimer’s disease, amnestic mild cognitive impairment, resting-state functional magnetic resonance imaging, degree centrality, voxel-mirrored homotopic connectivity, slow-5 frequency band

## Abstract

**Background:**

In the spectrum of Alzheimer’s Disease (AD) and related disorders, the resting-state functional magnetic resonance imaging (rs-fMRI) signals within the cerebral cortex may exhibit distinct characteristics across various frequency ranges. Nevertheless, this hypothesis has not yet been substantiated within the broader context of whole-brain functional connectivity. This study aims to explore potential modifications in degree centrality (DC) and voxel-mirrored homotopic connectivity (VMHC) among individuals with amnestic mild cognitive impairment (aMCI) and AD, while assessing whether these alterations differ across distinct frequency bands.

**Methods:**

This investigation encompassed a total of 53 AD patients, 40 aMCI patients, and 40 healthy controls (HCs). DC and VMHC values were computed within three distinct frequency bands: classical (0.01–0.08 Hz), slow-4 (0.027–0.073 Hz), and slow-5 (0.01–0.027 Hz) for the three respective groups. To discern differences among these groups, ANOVA and subsequent *post hoc* two-sample t-tests were employed. Cognitive function assessment utilized the mini-mental state examination (MMSE) and Montreal Cognitive Assessment (MoCA). Pearson correlation analysis was applied to investigate the associations between MMSE and MoCA scores with DC and VMHC.

**Results:**

Significant variations in degree centrality (DC) were observed among different groups across diverse frequency bands. The most notable differences were identified in the bilateral caudate nucleus (CN), bilateral medial superior frontal gyrus (mSFG), bilateral Lobule VIII of the cerebellar hemisphere (Lobule VIII), left precuneus (PCu), right Lobule VI of the cerebellar hemisphere (Lobule VI), and right Lobule IV and V of the cerebellar hemisphere (Lobule IV, V). Likewise, disparities in voxel-mirrored homotopic connectivity (VMHC) among groups were predominantly localized to the posterior cingulate gyrus (PCG) and Crus II of the cerebellar hemisphere (Crus II). Across the three frequency bands, the brain regions exhibiting significant differences in various parameters were most abundant in the slow-5 frequency band.

**Conclusion:**

This study enhances our understanding of the pathological and physiological mechanisms associated with AD continuum. Moreover, it underscores the importance of researchers considering various frequency bands in their investigations of brain function.

## 1 Introduction

Alzheimer’s Disease (AD), the most prevalent form of dementia among the elderly (Mckhann et al., 1984), entails irreversible neurodegeneration. Progressive decline in cognitive abilities and memory deterioration are typically associated with AD spectrum disorders, with memory being the primary affected function, followed by decline in other cognitive domains (Dubois et al., 2016). Presently, more than 6.7 million elderly individuals in the United States are afflicted by dementia, a figure projected to double by 2060 (Alzheimer's Disease Facts and Figures, 2023), posing significant healthcare challenges. AD is a continuum of biological and clinical manifestations, with amnestic mild cognitive impairment (aMCI) as its precursor, characterized by memory impairment and declining cognitive abilities, increasing the risk of AD dementia (Sperling et al., 2011). The transition from aMCI to clinically diagnosed AD remains inadequately understood, impeding the development of effective therapies (Sun et al., 2018). Investigating the progression of AD and aMCI is crucial for unveiling underlying mechanisms and advancing early interventions, potentially enhancing patient outcomes and alleviating societal burdens.

Resting-state functional magnetic resonance imaging (rs-fMRI) is a state-of-the-art, non-invasive method for assessing spontaneous fluctuations in blood oxygen level-dependent (BOLD) signals, which serves as a proxy for neural activity. Rs-fMRI techniques such as amplitude of low-frequency fluctuations (ALFF) (Xi et al., 2013), regional homogeneity (ReHo) (Zhang et al., 2012), and seed-based functional connectivity (FC) (Wang et al., 2006) have been extensively employed to investigate functional alterations in individuals with AD and aMCI. Unlike measures that focus on local areas like ALFF, ReHo, and subnetwork-scale seed-based FC, degree centrality (DC) offers an evaluation of the overall connectivity strength of the entire human brain on a global connectome scale. By identifying nodes with the highest number of direct connections, DC determines the relative importance of nodes within the network (Zuo et al., 2012). It has been utilized in the pathological and physiological exploration of AD and aMCI. A study (Xiong et al., 2021) discovered that, compared to healthy controls (HC), patients with MCI exhibit diminished DC values in the left inferior temporal gyrus. Additionally, these DC values exhibited positive correlations with Mini-Mental State Examination (MMSE) scores and negative correlations with disease progression. Another investigation (Li et al., 2018) revealed that individuals with subjective cognitive decline (SMC) demonstrate increased DC in both hippocampi (HP) and the left fusiform gyrus, along with decreased DC in the inferior parietal gyrus. This implies that DC can elucidate the inherent connectivity disruption patterns in the whole-brain functional network of AD patients at the voxel level.

However, in addition to intra-hemispheric connections, the functional connections between cerebral hemispheres are equally critical. Previous studies have indicated that AD patients may experience deficiencies in interhemispheric information integration (Lakmache et al., 1998). Therefore, it is advisable to employ voxel-mirrored homotopic connectivity (VMHC), an analytical approach detecting alterations in functional connectivity between homotopic regions across hemispheres (Zuo et al., 2010b), to delve further into the connectivity of homotopic regions and illuminate the underlying mechanisms of AD and aMCI. A study (Liao et al., 2018) discovered that the AD group exhibits lower VMHC values in the anterior cingulate gyrus and medial prefrontal cortex compared to the aMCI group and HC group. Utilizing rs-fMRI analysis grounded in both DC and VMHC can assess both whole-brain functional connectivity and interhemispheric connectivity. Examining the interplay between these two metrics may offer a more profound comprehension of the pathological mechanisms of AD.

Moreover, most investigations focusing on brain function in AD spectrum patients have centered on the classical frequency range (0.01–0.08 Hz) of rs-fMRI. However, research has indicated that rs-fMRI signals within the cortical regions of the brain manifest distinct characteristics and sensitivities to specific brain activity patterns across diverse frequency ranges (Buzsáki and Draguhn, 2004; Zuo et al., 2010a). This may relate to different neural representations, and previous study (Yuen et al., 2019) have already identified high reproducibility of these frequency clusters, regardless of the sampling rate of rs-fMRI data. Numerous studies (Han et al., 2011; Liu et al., 2014) have disclosed that in AD and aMCI patients, ALFF within the slow-5 frequency band (0.01–0.027 Hz) exhibits greater sensitivity to alterations in the default mode network (DMN) in comparison to the slow-4 frequency band (0.027–0.073 Hz). Given this, we previously investigated the discriminative capacity of the ALFF index in different frequency bands for aMCI patients and discovered that the slow-5 frequency band may offer insights into the pathogenesis of AD and its distinct stages (Wang et al., 2021). Nevertheless, this study did not delve deeper into whether individuals with AD spectrum disorders also display frequency-dependent characteristics in whole-brain functional connectivity. Hence, it is imperative to differentiate between frequency bands when examining alterations in DC and VMHC results in aMCI and AD patients.

In this study, we amassed rs-fMRI data to scrutinize disparities in DC and VMHC among the AD, aMCI, and HC groups. We also explored the association between variations in DC and VMHC in patients and neurocognitive assessments. More importantly, our study aims to ascertain whether DC and VMHC exhibit frequency band dependence in functional connectivity alterations among AD spectrum patients. This is the first study to apply DC and VMHC to evaluate multi-frequency band functional connectivity changes in patients with the AD spectrum. Building upon previous research, our hypothesis posits that aMCI and AD patients will manifest substantial abnormalities in DC and VMHC values relative to HC, and that these variations may correlate with disease severity. Additionally, we postulate that DC and VMHC within the slow-5 frequency band will demonstrate greater sensitivity to changes in functional connectivity in AD spectrum patients, in comparison to the slow-4 frequency band and the classical frequency range.

## 2 Methods

### 2.1 Participants

Between September 2016 and February 2018, a total of 61 AD and 52 aMCI patients were recruited from Zhejiang People’s Hospital, alongside 50 normal controls (NC) enlisted from the hospital health promotion center. All participants were right-handed and provided written informed consent before participating in the study. This prospective study received approval from the local Ethics Committee of Zhejiang Provincial People’s Hospital (Ethics Approval No. 2012KY002) and was conducted in accordance with the principles outlined in the Declaration of Helsinki. All participants underwent comprehensive assessments, including medical history collection, laboratory examinations, physical examinations, routine brain magnetic resonance scans, as well as Montreal Cognitive Assessment (MoCA) and Mini-Mental State Examination (MMSE) (Nasreddine et al., 2005) evaluations. AD patients were diagnosed based on the criteria outlined in the DSM-IV-R (revised Diagnostic and Statistical Manual of Mental Disorders, Fourth Edition) and the revised NINCDS-ADRDA (National Institute of Neurological and Communicative Disorders and Stroke and the Alzheimer’s Disease and Related Disorders Association) with an MMSE score of ≤24 (McKhann et al., 2011). The inclusion criteria for aMCI patients were as follows: 1) self-reported memory impairment; 2) absence of abnormal clinical manifestations; 3) MMSE score between 24 and 27 (McKhann et al., 2011); 4) failure to meet the criteria for dementia as defined by DSM-IV-R. NC participants were selected based on the following criteria: 1) the absence of neurological impairments such as visual loss or hearing loss, and 2) an MMSE score of ≥28. Patients and participants with a history of stroke, brain trauma, brain tumor, epilepsy, Parkinson’s disease, severe anemia, diabetes, hypertension, a history of mental illness, and signal abnormalities in the medial temporal lobe attributable to infectious or vascular factors detected in MRI FLAIR and T2-weighted images were excluded from the study.

### 2.2 Data acquisition

The MRI data were collected using a 3.0 T magnetic resonance scanner (Discovery MR750; GE Healthcare, Waukesha, WI, United States) at Zhejiang People’s Hospital. Anatomical images were acquired utilizing a high-resolution 3D T1-weighted magnetization-prepared rapid gradient echo (MPRAGE) sagittal sequence with the following parameters: repetition time (TR) = 6.7 ms, echo time (TE) = 2.9 ms, slice thickness = 1 mm, no gap, field of view (FOV) = 256 × 256 mm^2^, flip angle = 12°, in-plane resolution = 256 × 256, and 192 sagittal slices. The rs-fMRI images were captured using the echo-planar imaging sequence with the following settings: TR = 2,000 ms, TE = 30 ms, slice thickness = 3.2 mm, no gap, FOV = 220 × 220 mm^2^, flip angle = 90°, in-plane resolution = 64 × 64, comprising 210 volumes and 44 slices. Throughout the rs-fMRI data acquisition, all participants were instructed to remain still and keep their eyes closed, while staying awake.

### 2.3 Data preprocessing

The preprocessing of rs-fMRI data was executed utilizing the Data Processing and Analysis of Brain Imaging (DPABI 6.1) and Statistical Parametric Mapping 12 (SPM12) toolbox within the MATLAB 2018b platform (MathWorks, Natick, MA, United States). The preprocessing pipeline encompassed the following steps: (1) The initial ten volumes were discarded to ensure that the MRI signal reached a steady state, (2) Slice timing and head motion correction were applied to the remaining images, (3) The Diffeomorphic Anatomical Registration through Exponential Lie (DARTEL) technique was employed to construct the final tissue probability templates, based on the unified segmented images, (4) Functional images were normalized to the Montreal Neurological Institute (MNI) space with a resampling voxel size of 3 × 3 × 3 mm^3^, (5) Linear trend removal was conducted on the time course of the BOLD signal, (6) A noise removal process was implemented, encompassing the regression of Friston-24 head motion parameters, cerebrospinal fluid signals, and white matter signals, and (7) The band-pass filter was applied in the three frequency bands (classical frequency band: 0.01–0.08 Hz, slow-5 frequency band: 0.01–0.027 Hz, slow-4 frequency band: 0.027–0.073 Hz). Participants were excluded if head motion exceeded a 3-mm maximum displacement, rotation exceeded 3°, or the framewise displacement (FD) exceeded 0.5. Consequently, 133 participants (53 AD, 40 aMCI, and 40 NC) remained eligible for analysis.

### 2.4 DC calculation

To calculate the DC, Pearson’s correlation was applied to the time series between each voxel and all other voxels across the entire brain. This generated a correlation matrix, denoted as R = (rij), with j ranging from 1 to N-1 (where R represents the DC, r signifies the correlation coefficient of the given voxel, j corresponds to other voxels within the entire brain, and N denotes the number of voxels). A prior study demonstrated that different threshold selections did not qualitatively affect the results (Bao et al., 2021). The resulting matrices (DC maps) were smoothed with a Gaussian kernel (FWHM = 4 mm) to enable group comparisons and weighted graph calculation.

### 2.5 VMHC calculation

To generate VMHC maps, all normalized T1 images were averaged to create a mean normalized T1 image. Subsequently, a group-specific symmetrical template was established by averaging the left–right symmetric version of the mean image. The calculation of homotopic resting-state FC involved assessing the FC between any pair of symmetric inter-hemispheric voxels. The Pearson’s correlation coefficient was then computed between the residual time series of each voxel and its contralateral hemispheric counterpart. Finally, the resulting correlation values underwent Fisher Z-transform to standardize them. The resulting matrices (VMHC maps) were smoothed with a Gaussian kernel (FWHM = 4 mm) to enable group comparisons and weighted graph calculation.

### 2.6 Statistical analysis

Group differences in three DC and VMHC values (slow-5, slow-4, and classical frequency bands) were assessed within a gray matter mask using analysis of variance (ANOVA). We regressed four covariates: age, sex, education level, and head movement. The resulting F-maps were thresholded with a voxel-level threshold of *p* < 0.005 and a cluster-level threshold of *p* < 0.05, employing Gaussian Random Field theory (GRF) correction for multiple comparisons. Subsequently, *post hoc* comparisons were conducted in these clusters exhibiting significant group differences, employing the SPSS (SPSS Inc., Chicago, IL, United States) software. To correct for multiple comparisons, the Bonferroni correction procedure (*p* < 0.005) was applied.

For any cluster demonstrating among-group differences, a Pearson correlation analysis was employed to evaluate the association between the average DC and VMHC values of this cluster and MMSE and MoCA scores in all patients (aMCI and AD), with age, gender, and education serving as covariates of non-interest. Once again, the Bonferroni method (*p* < 0.005) was utilized to correct for multiple comparisons.

## 3 Results

### 3.1 Demographics and neuropsychological tests

A total of 53 patients with AD, 40 patients with aMCI, and 40 HCs were ultimately included in the data analysis. Notably, no significant differences in age (*p* > 0.05), gender (*p* > 0.05), or education level (*p* > 0.05) were observed among the AD, MCI, and HC groups (Table 1). However, the MMSE (*p* < 0.001) and MoCA (*p* < 0.001) scores exhibited significant differences across the three groups (Table 1). Furthermore, *post hoc* analysis revealed that significant differences in MMSE and MoCA scores were evident between each pair of groups (*p* < 0.001).

**Table 1:** Demographics and clinical characteristics of the participants.

Sample Size	AD (*N* = 53)	MCI (*N* = 40)	NC (*N* = 40)	*p*-value	*Post hoc* ^c^
Gender (male: female)	20:33	23:17	19:21	0.166^a^	–
Age (years, mean ± SD)	66.83 ± 7.85	66.23 ± 8.31	65.85 ± 9.18	0.851^b^	–
Education (years, mean ± SD)	7.36 ± 4.36	7.35 ± 3.05	7.38 ± 3.38	1.000^b^	–
MMSE	18.72 ± 4.54	26.28 ± 0.88	28.95 ± 0.90	<0.001^b^	NC > MCI > AD
MoCA	14.09 ± 5.49	22.65 ± 2.33	27.35 ± 1.35	<0.001^b^	NC > MCI > AD

^a^*p*-values for sex distribution obtained by the chi-square test; ^b^*p*-value obtained by analysis of variance; ^c^*Post hoc* testing obtained by Bonferroni correction.

AD, Alzheimer’s disease; aMCI, amnestic mild cognitive impairment; NC, normal controls.

ANOVA results across the three groups in three frequency bands.

### 3.2 DC index

**Figure 1:**
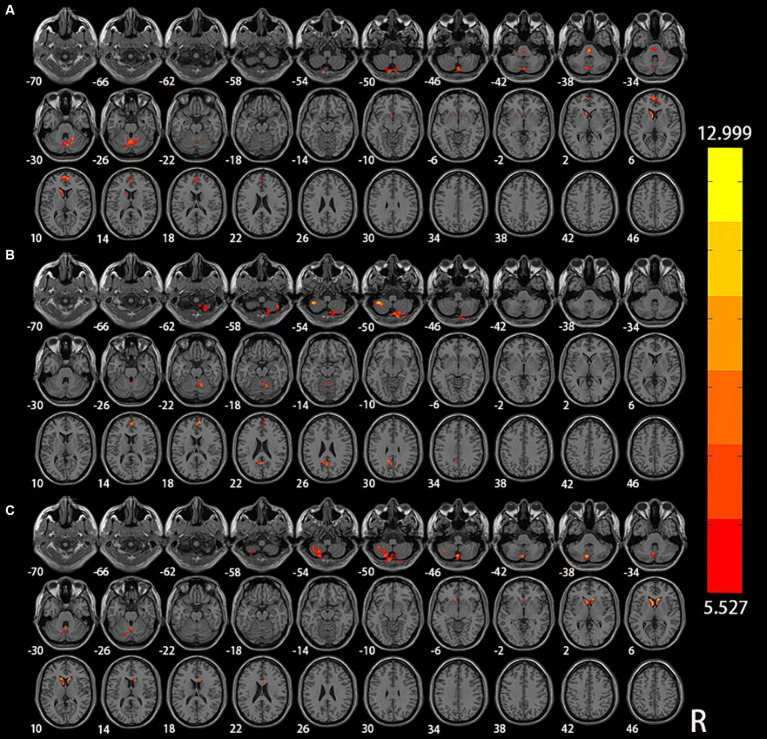
Significant differences in DC among NC, aMCI, and AD in different frequency bands: **(A)** classical frequency band; **(B)** slow-5 frequency band; **(C)** slow-4 frequency band.

**Figure 2:**
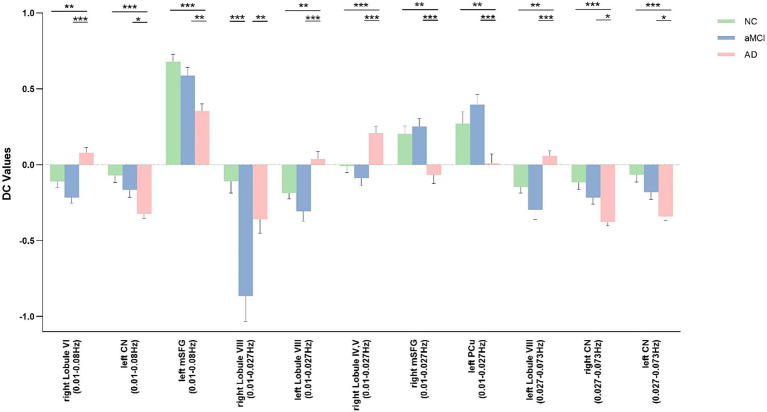
*Post hoc* comparisons of analysis of variance. The connection between two bars represents significant between-group differences (* indicates a significant level of *p* < 0.05, ** denotes a significant level of *p* < 0.01, and *** indicates a significant level of *p* < 0.001, Bonferroni correction). AD, Alzheimer’s disease; aMCI, amnestic mild cognitive impairment; NC, normal controls; Lobule VI, Lobule VI of the cerebellar hemisphere; CN, caudate nucleus; mSFG, medial superior frontal gyrus; Lobule VIII, Lobule VIII of the cerebellar hemisphere; Lobule IV, V, Lobule IV, V of the cerebellar hemisphere; mSFG, medial superior frontal gyrus; PCu, precuneus.

The results of the analysis of variance and subsequent *post hoc* analysis among the three groups within different frequency bands are illustrated in Figures 1, 2. Clusters displaying significant between-group differences were selected, and their anatomical locations, MNI coordinates, and peak values are presented in Table 2.

**Table 2:** Brain areas changed by DC across the three groups in three frequency bands.

Band	Cluster	Anatomical location	Peak MNI Coordinates			Peak intensity	AD vs. aMCI	AD vs. NC	aMCI vs. NC	All groups
			X	Y	Z					
Classical frequency	266	Right Lobule VI	21	−51	−30	9.261	<0.001	0.001	0.250	<0.001
	63	Left CN	−6	9	6	10.061	0.023	<0.001	0.816	<0.001
	108	Left mSFG	−3	60	9	9.0269	0.004	<0.001	0.321	<0.001
Slow-5 frequency	185	Right Lobule VIII	6	−84	−48	10.271	0.005	0.462	<0.001	<0.001
	48	Left Lobule VIII	−42	−48	−51	10.030	<0.001	0.006	0.338	<0.001
	52	Right Lobule IV, V	6	−54	−24	8.425	<0.001	<0.001	1.000	<0.001
	48	Right mSFG	9	57	18	9.182	<0.001	0.008	0.843	<0.001
	77	Left PCu	0	−57	24	9.890	<0.001	0.001	0.937	<0.001
Slow-4 frequency	308	Left Lobule VIII	−6	−75	−39	11.922	<0.001	0.001	0.209	<0.001
	71	Right CN	9	24	6	12.999	0.021	<0.001	0.163	<0.001
	72	Left CN	−6	9	6	11.415	0.017	<0.001	0.256	<0.001

*p*-values were obtained by Bonferroni-corrected post-hoc tests of multivariate ANCOVA adjusting for age, sex and education.

MNI, Montreal Neurological Institute; Lobule VI, Lobule VI of the cerebellar hemisphere; CN, caudate nucleus; mSFG, medial superior frontal gyrus; Lobule VIII, Lobule VIII of the cerebellar hemisphere; Lobule IV, V, Lobule IV, V of the cerebellar hemisphere; PCu, precuneus.

In the classical frequency band, notable inter-group differences were observed in the right Lobule VI of the cerebellar hemisphere (Lobule VI), left caudate nucleus (CN), and left medial superior frontal gyrus (mSFG) (voxel *p* < 0.005, cluster *p* < 0.05, GRF correction, cluster size >50 voxels). Compared to the NC and aMCI groups, the AD group exhibited increased DC in the right Lobule VI, left CN, and left mSFG.

Within the slow-5 frequency band, significant inter-group differences were evident in the bilateral Lobule VIII of the cerebellar hemisphere (Lobule VIII), right Lobule IV and V of the cerebellar hemisphere (Lobule IV, V), right mSFG, and left precuneus (PCu) (voxel p < 0.005, cluster p < 0.05, GRF correction, cluster size >38 voxels). In comparison to the NC and aMCI groups, the AD group demonstrated increased DC in the left Lobule VIII, right Lobule IV and V, right mSFG, and left PCu. Additionally, compared to the NC and AD groups, the aMCI group exhibited increased DC in the right Lobule VIII.

Within the slow-4 frequency band, significant inter-group differences were detected in the left Lobule VIII and bilateral CN (voxel *p* < 0.005, cluster *p* < 0.05, GRF correction, cluster size >45 voxels). In comparison to the NC and aMCI groups, the AD group displayed increased DC in the left Lobule VIII and bilateral CN.

### 3.3 VMHC index

**Figure 3:**
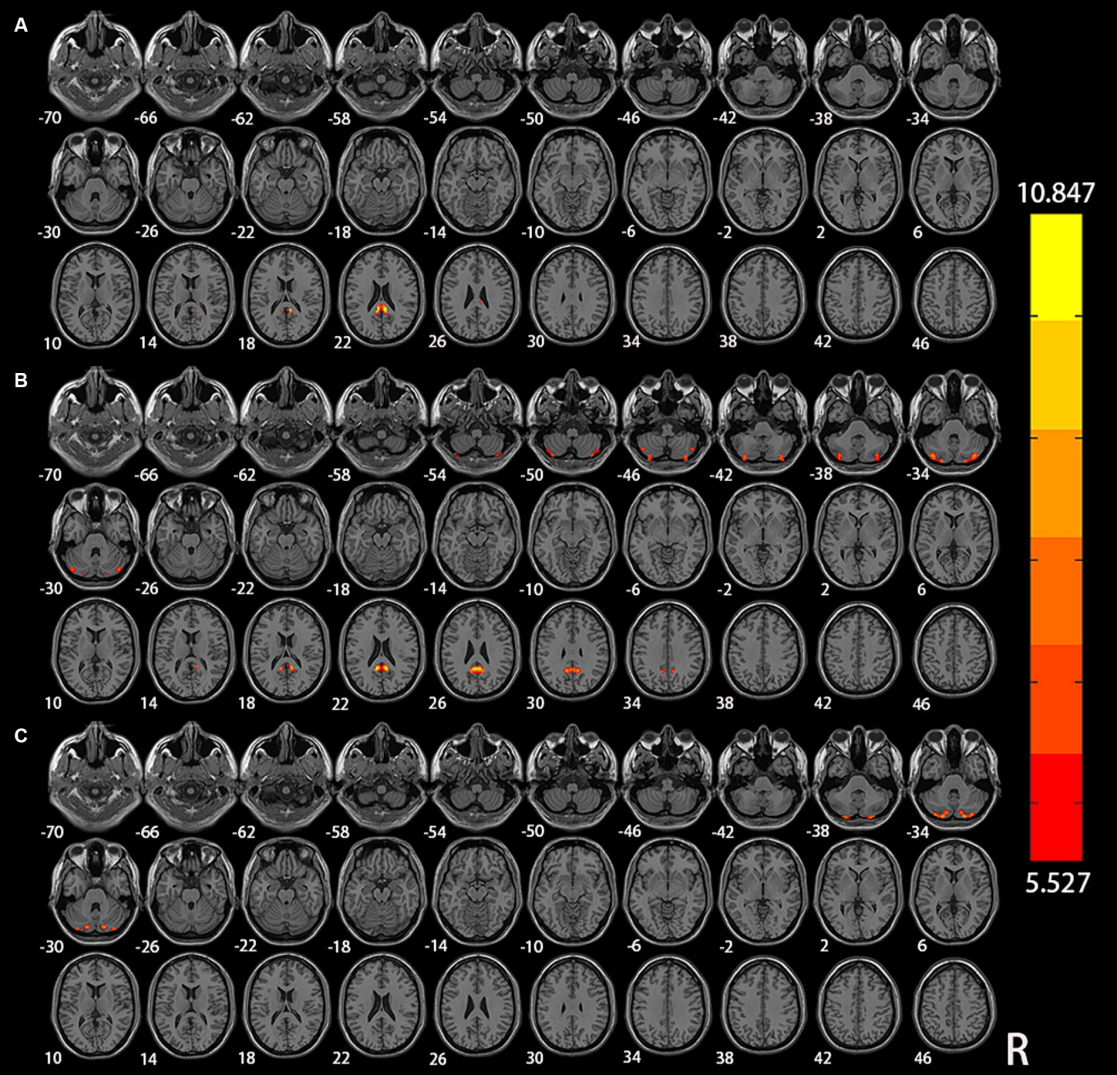
Significant differences of VMHC among NC, aMCI, and AD in different frequency bands: **(A)** classical frequency band; **(B)** slow-5 frequency band; **(C)** slow-4 frequency band.

**Figure 4:**
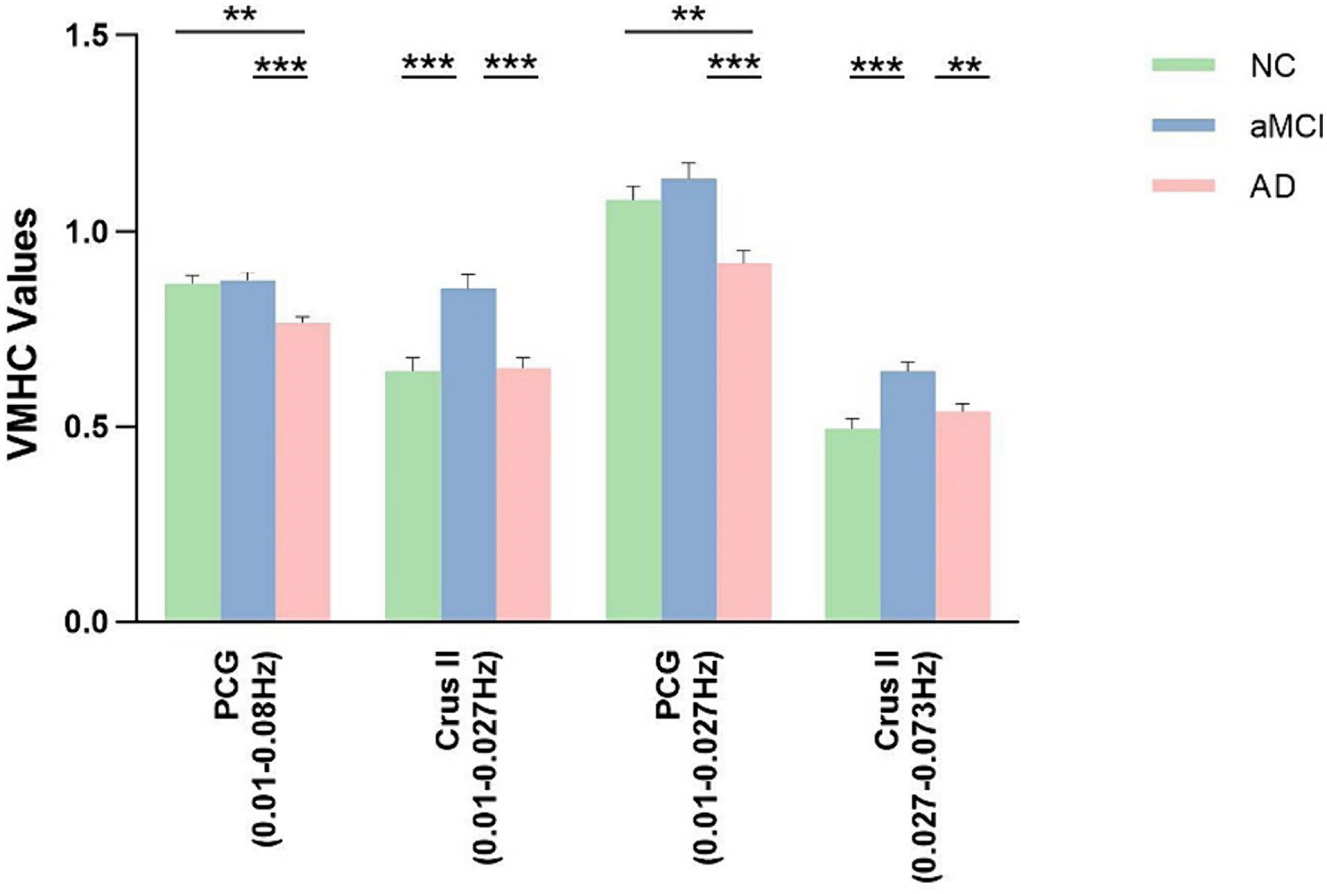
*Post hoc* comparisons of analysis of variance. The connection between two bars represents significant between-group differences (* indicates a significant level of p < 0.05, ** denotes a significant level of *p* < 0.01, and *** indicates a significant level of *p* < 0.001, Bonferroni correction). AD, Alzheimer’s disease; aMCI, amnestic mild cognitive impairment; NC, normal controls; PCG, posterior cingulate gyrus; Crus II, Crus II of the cerebellar hemisphere.

The results of the analysis of variance and subsequent *post hoc* analysis among the three groups within different frequency bands are depicted in Figures 3, 4. Clusters displaying significant between-group differences were selected, and their anatomical locations, MNI coordinates, and peak values are detailed in Table 3.

**Table 3:** Brain areas changed by VMHC across the three groups in three frequency bands.

Band	Cluster	Anatomical location	Peak MNI coordinates			Peak intensity	AD vs. aMCI	AD vs. NC	aMCI vs. NC	All groups
			X	Y	Z					
Classical frequency	102	PCG	±9	−42	21	10.847	<0.001	0.001	1.000	<0.001
Slow-5 frequency	265	Crus II	±39	−78	−33	10.594	<0.001	1.000	<0.001	<0.001
	198	PCG	±6	−48	24	8.089	<0.001	0.004	0.833	<0.001
Slow-4 frequency	151	Crus II	±21	−90	−36	9.660	0.002	0.589	<0.001	<0.001

*p*-values were obtained by Bonferroni-corrected post-hoc tests of multivariate ANCOVA adjusting for age, sex and education.

MNI, Montreal Neurological Institute; PCG, posterior cingulate gyrus; Crus II, Crus II of the cerebellar hemisphere.

In the classical frequency band, substantial inter-group differences were observed in the posterior cingulate gyrus (PCG). Relative to the NC and aMCI groups, the AD group exhibited increased VMHC in the PCG (voxel *p* < 0.005, cluster *p* < 0.05, GRF correction, cluster size >75 voxels).

Within the slow-5 frequency band, notable inter-group differences were observed in the PCG and Crus II of the cerebellar hemisphere (Crus II) (voxel *p* < 0.005, cluster *p* < 0.05, GRF correction, cluster size >72 voxels). In comparison to the NC and aMCI groups, the AD group displayed increased VMHC in the PCG. Additionally, compared to the NC and AD groups, the aMCI group exhibited increased VMHC in Crus II.

Within the slow-4 frequency band, significant inter-group differences were detected in Crus II (voxel p < 0.005, cluster *p* < 0.05, GRF correction, cluster size >67 voxels). In comparison to the NC and AD groups, the aMCI group demonstrated increased VMHC in Crus II.

### 3.4 Correlations between neuropsychological test and DC and VMHC values

**Figure 5:**
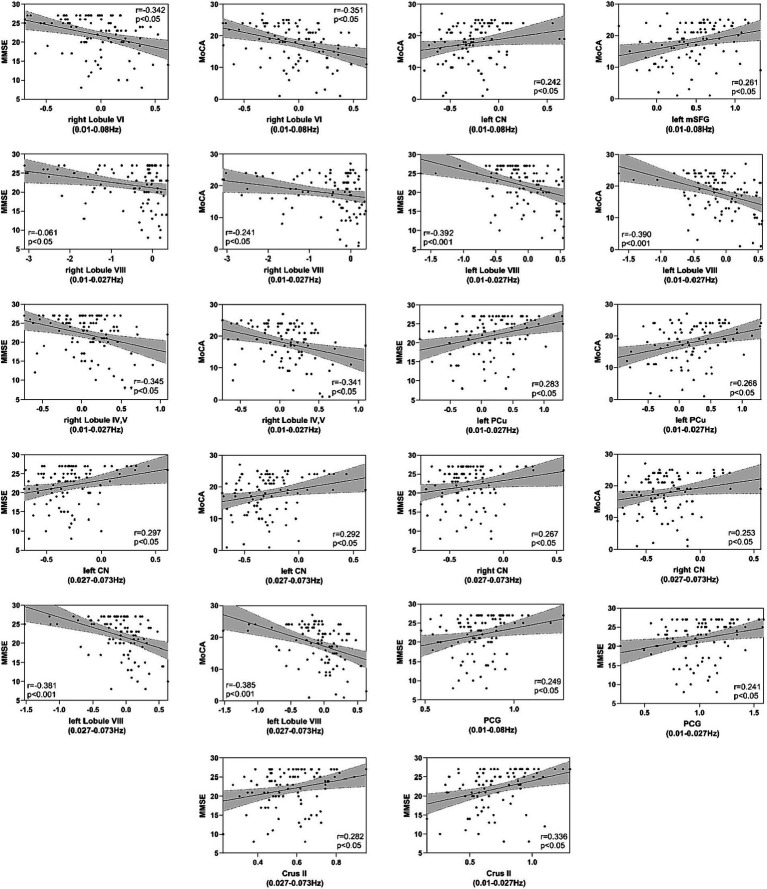
Correlation between MMSE and MoCA scales and DC and VMHC values in patients. The y-axis represents MMSE (Mini-Mental State Examination) or MoCA (Montreal Cognitive Assessment) scores, while the x-axis represents DC or VMHC values.

The correlations between the DC and VMHC values of each cluster representing inter-group differences and the MMSE and MoCA scores in patients within each frequency band are presented in Tables 4, 5 and Figure 5. For the DC values, clusters in bilateral CN and the left PCu exhibited positive correlations with MMSE, whereas clusters in bilateral Lobule VIII, right Lobule VI, and right Lobule IV, V demonstrated negative correlations with MMSE (*p* < 0.05, Bonferroni corrected). Similarly, DC values in clusters of bilateral CN, left PCu, and left mSFG displayed positive correlations with MoCA, while clusters in bilateral Lobule VIII, right Lobule VI, and right Lobule IV, V exhibited negative correlations with MoCA (*p* < 0.05, Bonferroni corrected). Regarding VMHC values, clusters in PCG and Crus II exhibited positive correlations with MMSE (*p* < 0.05, Bonferroni corrected).

**Table 4:** Correlation between DC value of each cluster showed among-group differences and MMSE and MoCA in different frequency bands.

Neuropsychological test	Bands	Brain regions	r	*p*-value
MMSE	Classical frequency	Right Lobule VI	−0.342	<0.05
	Slow-5 frequency	Right Lobule VIII	−0.061	<0.05
		Left Lobule VIII	−0.392	<0.001
		Right Lobule IV, V	−0.345	<0.05
		Left PCu	0.283	<0.05
	Slow-4 frequency	Left CN	0.297	<0.05
		Right CN	0.267	<0.05
		Left Lobule VIII	−0.381	<0.001
MoCA	Classical frequency	Right Lobule VI	−0.351	<0.05
		Left CN	0.242	<0.05
		Left mSFG	0.261	<0.05
	Slow-5 frequency	Right Lobule VIII	−0.241	<0.05
		Left Lobule VIII	−0.390	<0.001
		Right Lobule IV, V	−0.341	<0.05
		Left PCu	0.268	<0.05
	Slow-4 frequency	Left CN	0.292	<0.05
		Right CN	0.253	<0.05
		Left Lobule VIII	−0.385	<0.001

Lobule VI, Lobule VI of the cerebellar hemisphere; CN, caudate nucleus; mSFG, medial superior frontal gyrus; Lobule VIII, Lobule VIII of the cerebellar hemisphere; Lobule IV, V, Lobule IV, V of the cerebellar hemisphere; mSFG, medial superior frontal gyrus; PCu, precuneus.

**Table 5:** Correlation between VMHC value of each cluster showed among-group differences and MMSE in different frequency bands.

Neuropsychological test	Bands	Brain regions	r	*p*-value
MMSE	Classical frequency	PCG	0.249	<0.05
	Slow-5 frequency	Crus II	0.336	<0.05
		PCG	0.241	<0.05
	Slow-4 frequency	Crus II	0.282	<0.05

PCG, posterior cingulate gyrus; Crus II, Crus II of cerebellar hemisphere.

## 4 Discussion

To the best of our knowledge, this study represents the first application of DC and VMHC to assess FC changes in AD and aMCI patients across multiple frequency bands. This approach addresses a limitation observed in most studies in this field, which typically focus solely on the classical frequency bands. We have identified significant differences in DC/VMHC across various brain regions, including the right CN (slow-4), left CN (classical, slow-4), right mSFG (slow-5), left mSFG (classical), left PCu (slow-5), right Lobule VI (classical), right Lobule VIII (slow-5), left Lobule VIII (slow-5, slow-4), right Lobules IV, V (slow-5), PCG (classical, slow-5), and the Crus II (slow-5, slow-4) when comparing AD and aMCI patients to the control group. Furthermore, alterations in DC/VMHC in the bilateral CN, left PCu, right Lobule VI, left Lobule VIII, right Lobules IV, V, PCG, and the Crus II were found to be closely linked to the clinical characteristics of AD and aMCI patients. Most notably, we observed that the intergroup differences in the slow-5 frequency band were more pronounced than those in the classical and slow-4 frequency bands. These findings suggest that patients within the AD continuum exhibit abnormal FC changes in intrinsic brain activity, influenced by specific frequency bands.

DC, as a graph-based measure, reflects the number of direct connections of a given voxel within the entire brain connectivity matrix, thereby representing its centrality in the whole brain network. This method has been employed in research on various disorders, including AD (Wang et al., 2019), schizophrenia (Wheeler et al., 2015), Parkinson’s disease (Baggio et al., 2014), among others.

In this study, we observed a progressive decrease in the DC values of the bilateral CN and the left mSFG as the disease advanced from NC to aMCI and ultimately to AD. This trend indicates a continuous reduction in the direct connectivity of these regions within the entire brain as the disease progresses. The CN plays a crucial role in cognitive processes such as attention, planning, and the execution of complex goal-directed behavior (Cummings, 1995). It integrates primary inputs from the dorsolateral prefrontal cortex and the orbitofrontal cortex. Lesions in the CN can lead to deficits in executive control and cognitive processing speed. Previous research has linked progressive atrophy of the CN (Rombouts et al., 2000), as well as the deposition of tau and amyloid-beta protein (Braak and Braak, 1990), to AD. Our study provides whole-brain functional connectivity evidence to corroborate the significant role of the CN in the underlying mechanisms of AD. We speculate that as tau proteins and β-amyloid proteins continue to accumulate in the CN, the connectivity between the CN and the dorsolateral prefrontal cortex and orbitofrontal cortex decreases, leading to cognitive impairment symptoms associated with AD. The mSFG, located in the frontal lobe, is closely associated with cognitive functioning, information encoding and retrieval, emotional thinking, and perception (Jobson et al., 2021). It has strong connections with the anterior part of the prefrontal cortex and plays a critical role in human memory processing. Multiple studies (Yue et al., 2015; Cai et al., 2017) have consistently reported decreased FC in the mPFC of individuals with aMCI, particularly in connections with the hippocampus. As the disease progresses to AD, this reduction in functional connectivity becomes more pronounced (Gili et al., 2011). These prior studies lend support to our current research findings. The decrease in DC value in the mSFG indicates a reduced direct connectivity between this brain region and the anterior part of the prefrontal cortex and the hippocampus, which is believed to be associated with the weakened cognitive control, decreased intelligence, and lowered cognitive abilities in AD patients. Therefore, we speculate that the decreased neural activity in the frontal lobe region of AD patients may be related to the deterioration of memory function. Furthermore, when compared to the HC and aMCI groups, the AD group exhibited significantly decreased DC values in the left PCu and right mSFG. The PCu, situated on the medial surface of the parietal lobe, is associated with various higher-level cognitive functions, including episodic memory, self-referential information processing, and aspects of consciousness. In patients with AD, the PCu is recognized as the region with the most prominent tau pathology deposition and neuroinflammation (Veitch et al., 2019). FDG-PET imaging studies (Strom et al., 2022) have revealed early regional hypometabolism in the PCu of AD patients. Dynamic FC studies (Zhao et al., 2022) have shown significantly reduced connectivity strength in the PCu in patients with aMCI and AD compared to the normal control group. Our research findings align with these previously reported results. We believe that the PCu may be one of the earliest regions to exhibit tau pathology deposition, leading to reduced perfusion and metabolism in this area. Consequently, cognitive processes in both resting state and cognitive activities are impacted in AD patients. Additionally, we found that in patients with AD and aMCI, the DC of the left PCu and bilateral CN positively correlated with MMSE and MoCA scores, while the DC of the left mSFG positively correlated with MoCA scores. These correlations suggest that the DC values in these brain regions are associated with patients’ cognitive function and can serve as predictors of disease progression.

Compared to the HC and aMCI groups, the AD group exhibited significant increases in DC values in the right lobule VI, left lobule VIII, and right lobules IV, V. This indicates an elevated status and role of the cerebellum in the whole-brain network. In addition to its role in coordinating fine motor functions, the cerebellum is involved in regulating cognitive functions such as attention, language, executive control, and emotion (Yang et al., 2012). Multiple studies have highlighted the crucial role of the cerebellum in the progression and pathogenesis of AD. From normal cognition to MCI and AD, there is a gradual decrease in cerebellar gray matter volume. A study on MCI converters revealed a sustained decline in DC values in the cerebellum compared to HC (Hu et al., 2021). The hypothesis is that the reduction in cerebellar DC may contribute to the improvement of cognitive and motor functions in MCI patients. Our research findings align with this perspective. In this study, we observed a significant increase in DC values in the right cerebellar lobule VI, left cerebellar lobule VIII, and right cerebellar lobules IV and V in patients with AD. Furthermore, in both AD and aMCI patients, the DC values in these cerebellar regions were negatively correlated with MMSE and MoCA scores. The increased DC in the cerebellum may indicate a worsening of cognitive function in AD patients. We speculate that the elevated status and role of the cerebellum in the whole-brain network would inevitably consume more neural resources, leading to a reduction in direct connections with other brain regions that are more beneficial for maintaining cognitive functions, such as the CN and mSFG mentioned earlier. Interestingly, our study found that compared to the AD and HC groups, the aMCI group exhibited a significant decrease in DC values in the right lobule VIII. This could possibly be a compensatory mechanism for maintaining cognitive function in patients with aMCI.

Interhemispheric functional connectivity is a common characteristic of the brain’s intrinsic functional networks (Wang et al., 2006). VMHC measures the integration of information between the two hemispheres by calculating the connectivity between each voxel in one hemisphere and its mirrored counterpart in the other hemisphere. Significant patterns of disrupted VMHC have been observed in various disorders, including depression (Wang et al., 2013), schizophrenia (Hoptman et al., 2012), and autism spectrum disorder (Anderson et al., 2011).

In our study, the AD group exhibited significantly reduced VMHC values in the PCG compared to the HC and aMCI groups. This indicates impaired functional connectivity within the hemisphere in the mentioned region for AD patients. The PCG is a key node in the structural and functional networks of the human brain. It is involved in processing episodic memory, working memory, and short-term memory, playing an important role in the progression of AD. Multiple studies have reported reductions in ALFF, ReHo, and FC in the PCG of AD patients (Zhang et al., 2021; Liao et al., 2022). Our study further highlights impaired functional connectivity within the PCG between brain hemispheres, and this impairment is positively correlated with disease progression. Subsequent correlation analysis confirmed this finding, demonstrating a positive correlation between the VMHC of the PCG and MMSE scores in the patient group. Along with the previously mentioned mSFG and PCu, the PCG constitutes core brain regions within the default mode network (DMN). The DMN is the most stable resting-state network, and its activity during periods of rest is crucial for memory consolidation in humans. Therefore, it is believed to have potential relevance to the development of AD (Dennis and Thompson, 2014). Both the reduced direct functional connectivity of mSFG and PCu across the whole brain and the disrupted functional connectivity between the two cerebral hemispheres of PCG can lead to the disruption of the DMN. The aberrant resting-state brain function of the DMN is a primary characteristic of AD patients.

Additionally, in comparison to the HC and AD groups, the aMCI group exhibited an increase in VMHC values in the Crus II, and these VMHC values in the patient group were positively correlated with MMSE scores. Some studies (Zhang et al., 2020) suggest that Crus II is associated with the DMN and the fronto-parietal network (FPN), among other cognitive networks, and it plays a crucial role in cognitive representations. Therefore, the increase in the VMHC value of Crus II is considered a compensatory phenomenon in aMCI patients to maintain their cognitive function within the cerebellar region. Before developing into AD, aMCI participants may be able to recruit additional neural resources in the Crus II region that is less affected or unaffected by the disease to compensate for the disrupted inter-hemispheric connectivity in the PCG region. Another study (McLaren et al., 2012) also found increased cerebellar activity in mild AD patients, which was positively correlated with improved memory.

Brain oscillations encompass a wide range of frequencies. Zuo et al. (2010a) divided the low-frequency oscillations (LFO) frequency band into slow-5 (0.01–0.027 Hz), slow-4 (0.027–0.073 Hz), slow-3 (0.073–0.198 Hz), and slow-2 (0.198 Hz-0.25 Hz). Slow-4 and slow-5 oscillations, primarily detected in gray matter, fall within the classical frequency range (0.01–0.08 Hz) and reflect spontaneous brain activity. The slow-2 and slow-3 frequency bands are mainly confined to white matter and are associated with respiratory and cardiac signal contamination (Cordes et al., 2001). Consequently, most brain function studies are conducted in the classical frequency range. However, an increasing number of studies have found that intrinsic activity varies across different frequency bands and exhibits frequency-dependent alterations in various brain disorders, such as major depression (Wang et al., 2018), alcohol dependence (Guo et al., 2019) childhood epilepsy (Jiang et al., 2020), and trigeminal neuralgia (Ge et al., 2024).

Our study reveals that different brain regions exhibit both overlapping and distinct changes in the same index across different frequency bands, and to some extent, they complement each other. This is particularly evident in VMHC, where the slow-5 frequency band precisely encompasses all the brain regions in the classical and slow-4 frequency bands. However, for DC, each frequency band has its specific set of brain regions. For example, right Lobule VI and left mSFG were only detected in the classical frequency band. The specific brain regions associated with the slow-5 frequency band include the right Lobule VIII, right Lobule IV, V, right mSFG, and left PCu. The right CN was only detected in the slow-4 frequency band. Left Lobule VIII overlaps in the slow-5 and slow-4 frequency bands, while left CN overlaps in the classical and slow-4 frequency bands. These overlapping brain regions may play a more stable and important role in the pathogenesis of AD. In future research, we should focus on analyzing these brain regions. The differences in brain regions included in the slow-5 frequency band were the greatest. These findings are consistent with our previous research (Wang et al., 2021) and suggest that the brain functions exhibit specific frequency and engagement patterns. The intrinsic brain activity in different frequency bands may have specific pathological significance. The slow-4 and slow-5 signals are distinct entities with different roles, with the slow-5 frequency band potentially providing better exploration of the pathological mechanisms underlying AD.

Several limitations must be addressed in our study. First of all, the absence of a biological diagnosis of AD based on CSF/PET biomarkers is the greatest limitation. In the following research, we should enhance the biological marker data to achieve better diagnosis of patients within the AD continuum. Secondly, our investigation aims to understand the transition from NC to aMCI and eventually to AD. However, our study is cross-sectional, so the next step should involve longitudinal investigations to examine the changes in DC and VMHC during disease progression. Thirdly, noise from respiration and heartbeat during the scanning process can affect the low-frequency amplitudes in the resting state. Currently, we can only partially mitigate the confounding effects of these underlying factors. In future research, we can consider employing synchronized gating techniques to synchronize the MRI acquisition with specific phases of the cardiac or respiratory cycle. Finally, this study did not integrate functional metrics with structural metrics to further analyze and explore their correlations. Nevertheless, functional changes often have underlying structural bases. For instance, alterations in the volume of the corpus callosum mediate changes in interhemispheric connectivity. Therefore, future studies should integrate structural investigations in participants to gain a better understanding of the relationship between brain structure and functional changes.

## 5 Conclusion

This study has demonstrated that the simultaneous utilization of DC and VMHC metrics allows for a more comprehensive insight into the functional abnormalities in the underlying brain networks of AD spectrum patients. Furthermore, we observed that brain regions exhibiting significant variations in various parameters were most pronounced within the slow-5 frequency band. Therefore, we hypothesize that brain function demonstrates a certain degree of frequency specificity, and the slow-5 frequency band holds the potential to provide deeper insights into the pathological and physiological mechanisms of AD.

## Data availability statement

The original contributions presented in the study are included in the article/supplementary material, further inquiries can be directed to the corresponding authors.

## Ethics statement

This prospective study received approval from the local Ethics Committee of Zhejiang Provincial People's Hospital (Ethics Approval No. 2012KY002) and was conducted in accordance with the principles outlined in the Declaration of Helsinki. All participants were right-handed and provided written informed consent before participating in the study.

## Author contributions

HH: Formal analysis, Investigation, Methodology, Writing – original draft, Writing – review & editing. LW: Methodology, Validation, Writing – original draft, Writing – review & editing. SA: Methodology, Software, Writing – original draft. XT: Methodology, Validation, Writing – original draft. QF: Software, Visualization, Writing – original draft. YM: Software, Writing – original draft. XG: Methodology, Writing – original draft. ZL: Conceptualization, Validation, Writing – review & editing. ZD: Conceptualization, Validation, Writing – review & editing.
